# Immobilized Enzymes in Biosensor Applications

**DOI:** 10.3390/ma12010121

**Published:** 2019-01-02

**Authors:** Hoang Hiep Nguyen, Sun Hyeok Lee, Ui Jin Lee, Cesar D. Fermin, Moonil Kim

**Affiliations:** 1 BioNanotechnology Research Center, Korea Research Institute of Bioscience and Biotechnology (KRIBB), 125 Gwahangno, Yuseong-Gu, Daejeon 34141, Korea; nguyenhoanghiep244@gmail.com (H.H.N.); tnsgur02@kribb.re.kr (S.H.L.); uijin@kribb.re.kr (U.J.L.); 2 Department of Nanobiotechnology, KRIBB School of Biotechnology, Korea University of Science and Technology (UST), 217 Gajeongno, Yuseong-Gu, Daejeon 34113, Korea; 3 Department of Biochemistry, College of Natural Sciences, Chungnam National University, 99 Daehangno, Yuseong-Gu, Daejeon 34134, Korea; 4 Department of Biology, College of Arts & Sciences, Tuskegee University, Tuskegee, AL 36830, USA; fermin_c@mytu.tuskegee.edu

**Keywords:** enzyme, immobilization, biosensor, immobilized enzyme, enzyme modification

## Abstract

Enzyme-based biosensing devices have been extensively developed over the last few decades, and have proven to be innovative techniques in the qualitative and quantitative analysis of a variety of target substrates over a wide range of applications. Distinct advantages that enzyme-based biosensors provide, such as high sensitivity and specificity, portability, cost-effectiveness, and the possibilities for miniaturization and point-of-care diagnostic testing make them more and more attractive for research focused on clinical analysis, food safety control, or disease monitoring purposes. Therefore, this review article investigates the operating principle of enzymatic biosensors utilizing electrochemical, optical, thermistor, and piezoelectric measurement techniques and their applications in the literature, as well as approaches in improving the use of enzymes for biosensors.

## 1. Introduction

A biosensor is an analytical device that functions to analyze a sample in the presence of a specific target analyte. Typically, a biosensor is constructed from a biological component, which, in other words, is called a molecular recognition element, and a physicochemical detector component or transducer. The recognition elements in a biosensor are immobilized onto the surface of transducers, and they are able to interact with target molecules without adding reagents into the sample solution. In operation, the specific interactions between the target analyte and the recognition elements would produce physicochemical changes on the transducer surface. The changes are then recognized by the transducer, and converted into measurable signals which then could be used to determine the amount of analyte that is present in the sample. Generally, biosensors are classified based on either the biological component used, such as enzymes, antibodies, nucleic acids, or cells, or by the type of transducer, such as electrochemical, optical, mass-based, or piezoelectric transducer. There is another classification method that relies on the mode of interactions between the analytes and the biological materials in a biosensor. They are of two types: catalytic biosensor, in which the interactions result in the formation of a new biochemical reaction product, and affinity biosensor, in which the interactions result in analyte binding onto the transducer surface.

**Figure 1:**
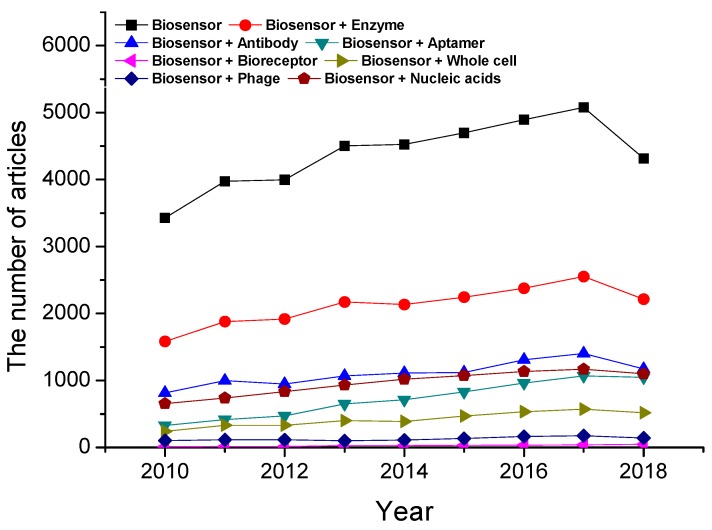
Trends in the annual numbers of published articles regarding bioreceptor-based biosensors (2010–2018). The horizontal axis represents the publication year, and the vertical axis shows the annual numbers of articles on scientific research.

Figure 1 shows the trends in the annual numbers of scientific articles covered by Scopus from 2010 to 2018 in the field of bioreceptor-based biosensors. Enzyme-based biosensors always rank first in the production of scientific articles, while the number of published articles associated with antibody-based biosensors rank second. In enzyme-based biosensors, the techniques of enzyme immobilization are highly significant, due to relative instability of the mobile enzyme, the difficulty in the active recovery of the enzyme, etc. [1]. On the other hand, the immobilized enzymes can be used repetitively and continuously, and maintain their catalytic activity with more stability than mobile enzymes [2]. In spite of a lower catalytic rate and additional treatment steps, the immobilized enzymes are widely utilized in medical and industrial areas, due to advantages such as rapid control by removing the enzymes, easy separation of the enzymes from the product, and high stability [3]. The approaches of the immobilized enzymes are various; adsorption, covalent bonding, entrapment, and cross-linking. First, adsorption is one of the most straightforward immobilization methods, and it is based on weak bonds such as Van der Waal’s forces, electrostatic, and hydrophobic interactions [4]. The advantage of the adsorption is simple and inexpensive, due to the non-necessity of an additional reagent, and it is less destructive to enzyme activity than other methods. However, the enzymes immobilized by this method are easily deposited by changes of experimental conditions, such as temperature, pH, or ionic strength, owing to their weak bonding [5]. Moreover, the non-specific adsorption of other substrates onto a surface may lead to contamination, and interference to the signal. Second, covalent bonding is one of the most widely used methods, and it offers stable complexes between enzymes and supports. The side chains, such as lysine (ε-amino group), cysteine (thiol group), and aspartic and glutamic acids (carboxylic group) in the enzyme are usually used for the formation of covalent bonding [6] and supports form a self-assembled monolayer (SAM) prior to a coupling reaction with enzymes. The covalently immobilized enzymes possess superiorly strong bindings over adsorption, so this method can provide more stably immobilized enzymes. The high uniformity of SAM and the good control of the immobilized enzyme amount are also advantages for covalent immobilization. Despite several strengths, the formation of covalent bonding affects the activity of the immobilized enzymes, and large amounts of bioreagent are required for this method. Third, entrapment is not directly attached, but entrapped in polymers, which retains a space where substrates and products freely disperse. Polymerization is carried out in a mixture of enzymes and monomers for the entrapment of enzymes. The entrapment is not chemical interaction, unlike covalent bonding, and gives the enzymes high stability and the minimization of leaching. However, the gel matrix can interfere with the deep diffusion of substrates to the active site of the enzyme, and this method also has a low loading capacity of the enzymes. Finally, the intermolecular cross-linkages between the enzymes form a three-dimensional enzyme complex through covalent bonding. To form cross-linking between enzymes, free amino groups of lysine residue in enzymes react with a reagent such as glutaraldehyde (GTA). The immobilization approach by cross-linking improves the efficiency and stability, because of highly strong and stable bonding between enzymes. However, the usage of cross-linking reagents such as GTA can lead to a loss of activity from severe modifications of the enzymes due to covalent bonding [3].

Biosensors that use immobilized enzymes belong to a catalytic type of biosensor in which the transducer surface is immobilized with enzymes that act as a bridge between the transducer and the analyte. For the immobilization benefits mentioned in the above paragraph, immobilized enzyme-based biosensors have been used widely in various fields of applications, such as in biomedical applications [7,8], the detection of environmental pollutants [9,10], food safety monitoring [11,12], and industrial bioprocess monitoring [13,14]. This review paper on the use of immobilized enzymes in biosensors, will begin with a section describing the types of enzyme-based biosensors. In the next section, approaches in improving the use of enzymes for biosensors will be discussed with regards to enzyme modification by biological and chemical methods, and the multi-enzyme systems used in biosensors.

## 2. Enzyme-Based Biosensors

A biosensor is generally made up of three components; a biological recognition element, a transducer, and a signal processing system. In an enzyme-based biosensor, the enzyme is utilized as the recognition element, and is immobilized on/within the support matrix on the transducer surface in order to maintain enzyme activity. The advantages of using enzymes, such as the high specificity of enzyme–substrate interactions and the high turnover rates of biocatalysts (i.e., the product of catalyst activity and lifetime), have made enzyme-based biosensors one of the most extensively studied areas. The sensing principle of the enzyme-based biosensor is to detect the presence of certain analytes by measuring changes such as: proton concentration (H^+^), the release or uptake of gases (i.e., CO_2_, NH_3_, O_2_, etc.), light emission, absorption or reflectance, heat emission, and so forth, which occurs during substrate consumption or product formation of an enzymatic reaction. The transducer then converts those changes into measurable signals (electrical, optical or thermal signals) that are used to identify analytes of interest. In this review paper, enzyme-based biosensors are divided into several categories based on the transducer types: electrochemical, optical, thermal/calorimetric, and piezoelectric biosensors.

### 2.1. Enzyme-Based Electrochemical Biosensors

Electrochemical biosensors are one of the most extensively used biosensors whose working mechanisms are based upon the electrochemical properties of transducers and analytes. Since the early 1960s, this type of biosensor has been developed after the first concept of the glucose enzyme was proposed [15]. Basically, changes in physicochemical properties of electroactive substances such as: current, voltage, resistance, or superficial charge, produced by redox reactions occurring on the transducer electrode surface, are output signals. The use of electrochemical biosensors provides advantages such as simplicity, rapidity, low cost, and high sensitivity. The most common types of transducers used in electrochemical biosensors are amperometry, potentiometry, conductometry, and impedimetry.

#### 2.1.1. Amperometric Biosensors

In an enzyme-based amperometric biosensor, the measured signal is the current, which is produced by the oxidation or reduction of electroactive species at the working electrode (i.e., gold, carbon, platinum, etc.). The magnitude of the current, which is produced at the surface of the working electrode, is proportional to the concentration of analytes present in the test solution upon addition of the substrate, and is monitored when a fixed potential is applied between the two electrodes. Enzyme-based amperometric biosensors have been widely studied, due to advantages such as ease in miniaturization, robustness, and the capability to operate with small sample volumes of rather complex matrices [16,17]. Amperometric enzyme biosensors have been developed through three generations, according to the electron transfer methods that have been used for the measurement of the biochemical reaction [18]. Nowadays, several kinds of commercial enzyme-based amperometric biosensors are accessible for measuring glucose, lactate, alcohol, etc., by using oxidases (i.e., glucose oxidase, lactate oxidase, alcohol oxidase, etc.) that oxidize their substrates producing hydrogen peroxide (H_2_O_2_) which is detected by the electrode.

**Figure 2:**
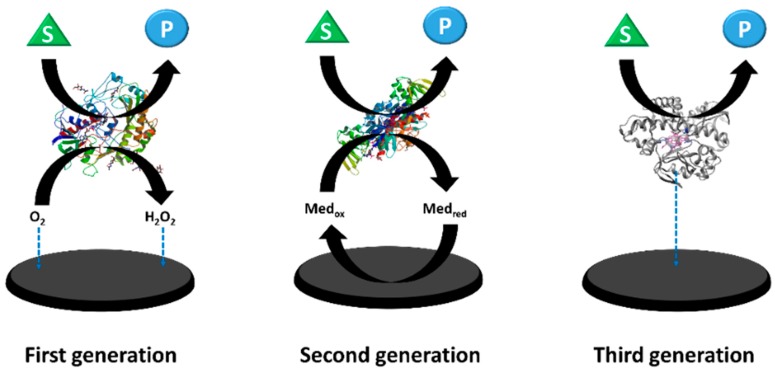
Schematic illustration of first-, second-, and third-generation amperometric enzymatic biosensors. First generation is based on the electroactivity of the receptor substrate or the product. Second generation is based on the use of artificial redox mediators. Third generation is based on the direct electron transfer between the redox-active biomolecule and the electrode. Adapted from [18].

Figure 2 shows a schematic representation of these different approaches in the development of enzyme-based amperometric biosensors. In a first-generation biosensor, the enzyme is immobilized onto a transducer surface and analyte substrate is monitored through the measurement of the enzymatic product (i.e., H_2_O_2_, NADH, etc.) or on the monitoring of the consumption of the co-factor, such as oxygen. The main advantages of first-generation amperometric biosensors are their high sensitivity and the low response time (around one second). However, drawbacks such as the requirement of coenzymes and the high potential for operation, fouling transducers surface due to prolonged use, and an error that relies on electron acceptor concentration (such as dissolved molecular oxygen concentration) may limit its usage in biological systems [18,19,20]. In order to eliminate oxygen dependency, second-generation biosensors use a mediator (electron acceptor), instead of oxygen, to transport the electrons to the electrode. The mediator is small redox active molecules (i.e., ferrocene derivatives, ferrocyanide, conducting organic salts, and quinones) that react with the enzyme-active site, and then with the electrode surface, thus transferring electrons to produce a current signal that is proportional to the detected analyte concentration. In this principle, the mediator is reduced by the electron generated from the enzymatic reaction, and then is finally oxidized at the electrode, resulting in electron transfer to the electrode. Although the second-generation biosensor is oxygen-independent, it suffers from mediator leaching and interference, due to redox mediator selectivity [18,19,21]. Hence, the third-generation biosensor was developed to solve that issue. In third-generation biosensors, the electron transfer between enzyme and electrode occurs in a direct manner without mediators or co-substrates during the catalytic transformation of the substrate to the product. The redox enzyme plays a role as an electrocatalyst that facilitates the electron transfer between the electrode and the substrate molecule. To improve the efficiency of the electron transfer, charge–transfer complexes such as tetrathiafulvalene–tetracyanoquinodimethane are often in use [22]. Third-generation biosensors offer superior selectivity because they are able to operate in a potential range that is closer to the redox potential of the enzyme [23,24]. Recently, an interesting study regarding an amperometric H_2_O_2_ biosensor based on artificial heme enzyme mimics by Palanisamy et al. was reported [25]. The researchers fabricated a novel H_2_O_2_ biosensor using a hemin-immobilized reduced graphene oxide–cellulose microfiber (RGO-CMF) composite. The sensor exhibited a linear response to hydrogen peroxide over a concentration range from 0.06 to 540.6 μM, with a lower detection limit of 16 nM. The RGO-CMF composite may be useful for the immobilization of redox active enzymes, and the fabrication of biosensors. It is for that reason that enzyme-catalyzed reactions are less exposed to interfering reactions. In addition, the sensitivity of this kind of biosensor can be also improved, due to higher integration between the biomolecule and the electrode surface than the previous generations.

#### 2.1.2. Potentiometric Biosensors

Since many enzyme reactions involve in the release or absorption of hydrogen ions, which result in the changes of ionic concentration, an ion-selective electrode could be used to monitor these processes. In a potentiometric biosensor, the difference in potential (voltage) between the working electrode and the reference electrode is considered as a signal that is measured under equilibrium conditions (in which there is no current flow), in order not to cause interference with the reaction. The measured signals form a function of target analyte concentrations in a logarithmic manner, and are used for quantification [26]. Potentiometric biosensor classification: Ion-selective electrode (ISE), enzyme field-effect transistor (EnFET) and light-addressable potentiometric sensor (LAPs).

##### Ion Selective Electrodes

An ion-selective electrode is used in a potentiometric sensor, in order to convert the activity of a specific ion in test solution into a voltage (potential), which can be measured by a pH/mV meter. The electrode is usually composed of two components: (1) An ion-specific membrane that provides a preference permeability for specific ions in the analyte solution, which contains a variety of interfering ions, and (2) a separate or integrated reference electrode. After ion penetration, an electrochemical equilibrium is established, and a difference in potentials is formed between the two phases (the reaction solution and the inner/measuring solution). Owing to the membrane specificity, this potential difference is controlled only by the activities of a specific ion in these phases. There are five main types of ion selective electrodes, which are classified by the nature of the membrane material used to construct the electrode; these are: glass membrane electrode, solid-state membrane electrode, polymer membrane electrode, gas-permeable membrane electrode, enzyme electrode. The differences in membrane construction are the features that make an electrode selective for a particular ion. Among the types of electrodes, the most commonly used electrodes in enzyme biosensors are gas-sensing electrodes and enzyme electrodes. Gas-sensing electrodes have gas-permeable membranes separating an enzyme reaction solution from an internal solution. When gas molecules diffuse across the gas-permeable membrane, they hydrolyze in the thin film of internal solution, leading to variations of some ion concentrations (generally H^+^), which results in a pH change that could be detected by a pH electrode. Therefore, the potential changes are directly related to the concentration of gas existing in the reaction solution. Common gas-sensing electrodes include membranes that are specific for ammonia, carbon dioxide, and nitrogen oxide gas.

**Figure 3:**
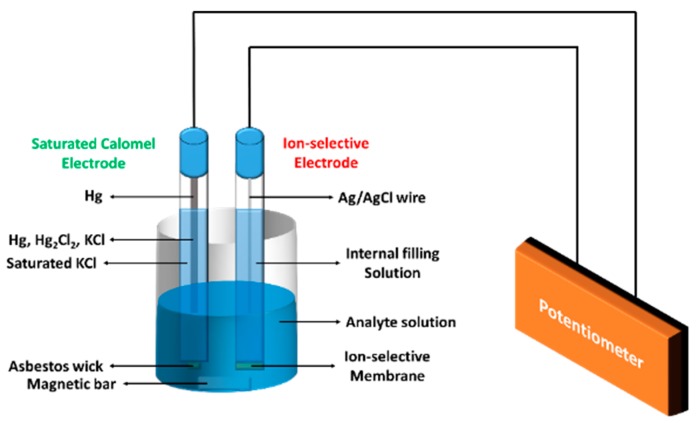
Schematic diagram of a conventional ion selective electrode. The electrochemical cell is made of Ag/AgCl|internal filling solution| polyvinyl chloride (PVC) membrane|analyte solution|Hg/Hg_2_Cl_2_, KCl (saturated). Adapted from [27].

Figure 3 shows a brief schematic illustration of an ion-selective electrode [27]. Potentiometric biosensors such as ISE can be useful for screening biological samples, due to the advantages of simple design, construction, operation, high selectivity, fast response time, and possible interfacing with automated systems [28]. Enzyme-based ion-selective electrodes are commonly fabricated by immobilizing an enzyme in a membrane that is coated on the surface of an appropriate electrode, such as a pH electrode or a gas-permeable membrane electrode, to monitor the reaction that occurs. For example, an enzyme electrode for β-d-glucose detection can be fabricated by immobilizing glucose oxidase onto either a pH electrode (which measures the liberated gluconic acid), a Pt electrode (which measures the H_2_O_2_ release or the O_2_ uptake), an iodide membrane electrode (which senses the I-uptake), or onto a gas membrane, or a Clark-type O_2_ electrode (which measures the O_2_ uptake). Among these options, the commonly chosen approach is to measure either the O_2_ uptake or the H_2_O_2_ release.

##### Enzyme Field-Effect Transistors 

An EnFET sensor is constructed based on an ion-sensitive field-effect transistor (ISFET), which is built by separating the metal gate of a classical MOSFET (metal oxide semiconductor field-effect transistor) from the device, and reconstructing the gate in the form of a reference electrode inserted in an aqueous solution, which is separated with the gate oxide by an enzymatic membrane [29]. When such ISFETs are coupled with the immobilization of a thin enzyme membrane at the gate surface, they become EnFETs. The types of membranes were relatively similar to ion-selective electrode classification, stated in the previous section. The EnFET sensor originates from a pH-sensitive detector, in which the enzymatic catalytic activity is sensitive to the pH level. Therefore, the resulting concentration of protons (H^+^) is directly proportional to that of the substrate. The enzyme-modified FET is operationally based on an enzymatic reaction where the enzyme specifically catalyzes the conversion of a substrate to its product. The EnFET device can be useful for both quantitative and qualitative measurement of enzyme–substrate reactions. These enzymatic reactions influence the presence of the accumulated charge carriers on the gate surface in proportion to the quantity of analyte present in a sample. The accumulation of charge carriers on the gate electrode occur according to the corresponding catalyzed reactions until the substrate molecules are used up. A measurable change in the electrical signal between the source (S) and the drain (D) can be caused by the enzymatic reactions. The most common EnFET device may be a FET sensor for glucose detection, in which the gate electrodes are modified with glucose oxidase to produce H_2_O_2_ according to: glucose + O_2_ + glucose oxidase → H_2_O_2_ + gluconic acid. The working principle of an EnFET is as follows: During the enzymatic reaction in the enzyme membrane, protons are generated or consumed, leading to a change in pH that could be measured by using a pH-electrode as a reference electrode [30]. The change of pH solution can be correlated to the analyte concentration. A large number of EnFET biosensors have been created after the first report for the determination of penicillin, in which the practical applicability of an ISFET as a pH-based EnFET was evaluated [31].

**Figure 4:**
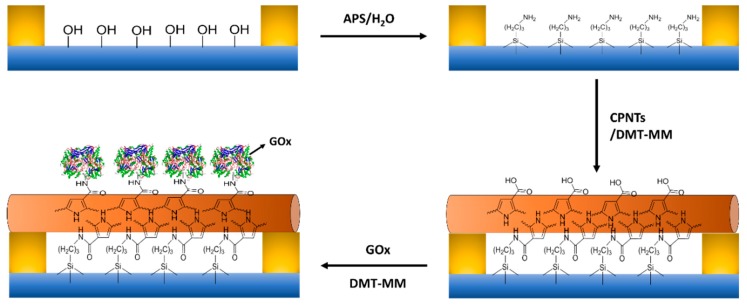
Schematic diagram of reaction steps for the fabrication of polypyrrole nanotube-based EnFET. Step 1: Microelectrode substrate; Step 2: Aminosilane-treated substrate; Step 3: Immobilization of the nanotubes onto a substrate; Step 4: Binding of glucose oxidase (GOx) to the nanotubes. Adapted from [32]. APS: 3-aminopropyltrimethoxysilane; CPNTs: carboxylated polypyrrole nanotubes; DMT-MM: 4-(4,6-dimethoxy-1,3,5-triazin-2-yl)-4-methylmorpholinium chloride.

Yoon et al. reported a liquid ion-gated EnFET sensor constructed on the basis of the chemical functionality of carboxylated polypyrrole nanotubes (CPNTs) for glucose detection [32]. Figure 4 shows a schematic reaction steps for the fabrication of enzyme-functionalized CPNTs. In that study, enzyme-functionalized polypyrrole nanotubes were used as the conductive channel, providing real-time response and high sensitivity. Also, MnO_2_ nanoparticle- and SiO_2_ nanoparticle-based EnFET sensors were developed for the detection of glucose [33,34]. So far, EnFETs have been developed for monitoring of a wide variety of anaytes such as urea [35,36], pesticides [37,38], phenolic compounds [39], steroidal glycoalkaloids [40], and creatinine [41]. The use of EnFET in sensing applications has certain advantages of miniaturization, excellent activity, multi-analyte detection potential, low cost, and high sensitivity; however, buffer compositions and conditions such as ionic strength, ionic concentration, buffer capacity, and buffer pH may greatly affect the sensor performance, as the sensor operation is heavily dependent on pH changes.

##### Light-Addressable Potentiometric Sensors (LAPS)

In LAPS, a modulated light from a light-emitting diode (LED) is used for semiconductor activation instead of applying an alternating current (AC) voltage. Under illumination, electron-hole pairs are generated on parts of the semiconductor surface. As a result, a photocurrent is produced and measured under a fixed bias voltage. The LAPS is a semiconductor-based chemical sensor which is built following the electrolyte–insulator–semiconductor (EISC) structure [42]. For example, a pH-sensitive LAPS that uses LEDs in combination with silicon as semiconductor and SiO_2_/Al_2_O_3_ as pH-sensitive insulator was developed to build an EISC-based sensor for the detection of urea, penicillin, and glucose [43]. LAPS was also used for the monitoring of enzyme activity and inhibitors. For example, biotinylated acetylcholinesterase (AChE (EC 3.1.1.7)) from eel was immobilized to a biotinylated cellulose nitrate membrane, and enzyme activity was determined as a function of substrate concentration and the amount of immobilized enzyme [44]. The sensor was able to quantify concentrations of substrate such as acetylcholine diisopropylfluorophosphate (DFP) and echothiophate in the range of 1–10 ppb. In addition, LAPS was also verified for its capability to detect numerous other insecticides [45].

**Figure 5:**
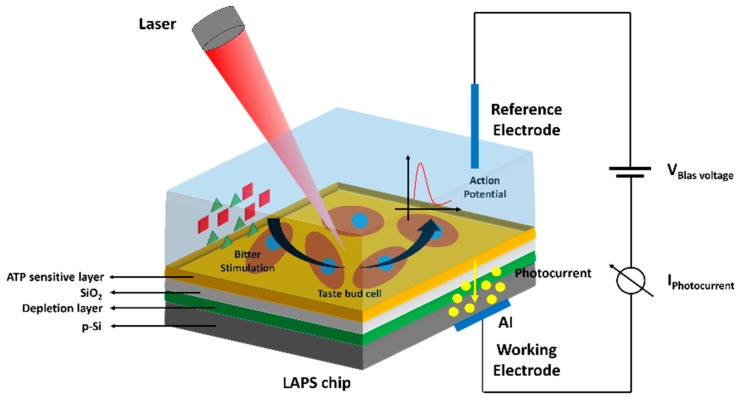
Schematic diagram showing the mechanisms of the LAPS chip for the extracellular recording of cell membrane potential changes of single taste bud cells in response to bitter substances. Adapted from [49]. LAPS: light-addressable potentiometric sensor.

The LAPS sensor is a photoelectric semiconductor that is sensitive to surface potential changes. Thus, responses that lead to the changes of surface potential, such as cell membrane potential changes [46], ion concentrations [47], and charged molecules [48] can be measured by using LAPS. Recently, Du et al. reported the extracellular recording of adenosine triphosphate (ATP) release via a LAPS chip functionalized with an ATP-sensitive DNA aptamer (Figure 5) [49]. In that study, changes in extracellular membrane potential were also monitored by recording the fluctuation of the LAPS photocurrent. The LAPS biosensor can be useful for investigating biological signal transduction at the single-cell level.

#### 2.1.3. Conductometry

Enzymatic reactions often involve changes in the ionic concentration, which alter the electrical conductivity of the electrolyte solution. This solution conductivity can be measured through a conductometric biosensor by applying a potential difference between two parallel electrodes. As a consequence, ion mobility is increased due to the movement of negatively charged ions toward the anode, and positively charged ions toward the cathode. The conductivity of the electrolyte solution solely depends on the ion concentration and mobility, thus this measurement could be useful in cases where there are none or negligible electrochemical reactions occurring on the electrodes. In a similar manner to other electrochemical based biosensors, the principles and methods of enzyme immobilization on electrodes for an amperometric biosensor are suitable for conductometric transducers. For example, the electrode surface was immobilized, with the enzyme included inside an albumin gel film by the means of covalent attachment using glutaraldehyde [50]. Also, the enzyme was immobilized on an electrode surface by using the sol-gel entrapment method [51], covalent binding with a collagen membrane [52], electrochemical polymerization [53], or cross-linking with bovine serum albumin using glutaraldehyde [54]. Table 1 summarizes the types of substrates and immobilized enzymes, and the immobilization method for conductometric enzyme biosensors.

#### 2.1.4. Impedimetric Enzyme Biosensors

In an electrochemical impedimetric-based biosensor, the impedance of the electrode is the measurable response. Electrochemical impedance spectroscopy (EIS) is employed for investigating the changes in interfacial properties, owing to bio-recognition events occurring at the modified surfaces. The obtained impedance spectrum could then be used to determine quantitative parameters of electrochemical processes. In enzyme-based biosensors, this impedance measurement technique is less frequently used in comparison with potentiometric and amperometric techniques, due to the time-consumption in the record of a full impedance spectrum within a broad region of frequencies. In addition, in the EIS technique, several requirements such as linearity, stability, and causality are met to obtain a valid impedance spectrum. Hence, EIS techniques are commonly used as characterizable methods for most of the enzyme-based impedimetric biosensors. Shervedani et al. developed an impedimetric biosensor for the determination of glucose based on EIS measurements [70]. In this method, glucose oxidase (GOx (EC 1.1.3.4)) was immobilized onto the SAM of a mercaptopropionic acid (MPA)-modified gold electrode (Au-MPA-GOx SAMs). Parabenzoquinone (PBQ) was used as an electron mediator that is reduced to hydroquinone (H_2_Q), which in turn is oxidized at the Au electrode in the diffusion layer. The EIS measurements showed that the increase in the glucose concentration corresponds to a decrease in the faradaic charge transfer resistance (Rct) as a result of an increase in the diffusion current density of the H_2_Q oxidation. Glucose is quantified according to a linear function of sensor responses (1/Rct) and glucose concentration in solution. The nondestructive and straightforward method showed a dynamic range of glucose determination, with a detection limit of 15.6 μM. Recently, Zehani et al. reported a new impedimetric biosensor system devoted to environmental applications, utilizing immobilized lipase from *Candida rugosa* (a CRL-microbial source) and lipase from porcine pancreas (PPL-animal source) for the detection of diazinon in an aqueous medium [71]. The bioselective enzyme membranes were fabricated by the functionalization of gold microelectrodes with a SAM of thioacid, and the enzyme and bovine serum albumine (BSA) were cross-linked by using glutaraldehyde (GA). Upon increasing the concentrations of diazinon, the CRL biosensor showed total impedance decreases, from 2 to 50 μM. A saturation effect is observed for diazinon concentrations of higher than 50 μM. The two biosensors using two types of lipase could both be used for diazinon detection in a wide range of linearity of up to 50 μM, with a detection limit of 10 nM for the CRL biosensor, and 0.1 μM for the PPL biosensor. In addition, the sensors showed good accuracy and reproducibility, as well as good storage and stability for 25 days under 4 °C storage conditions.

**Figure 6:**
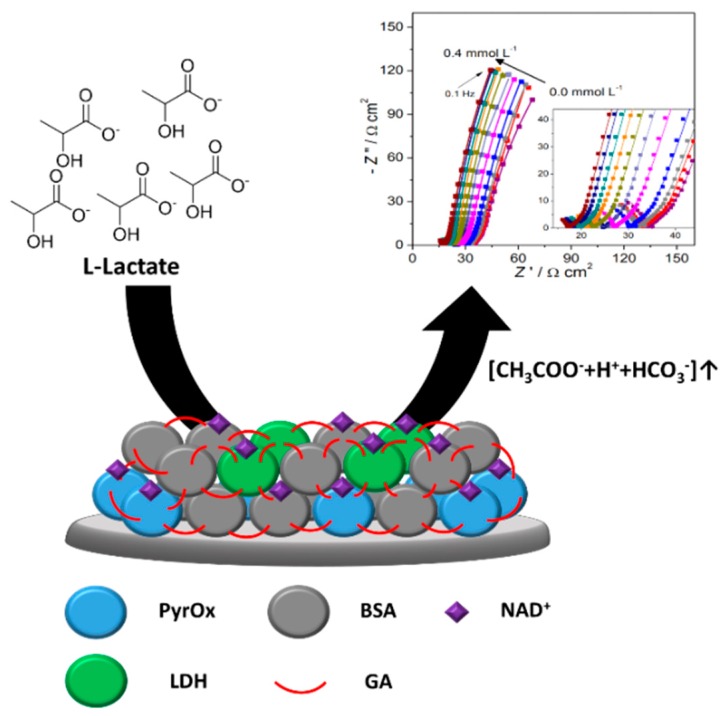
Schematic representation of an l-lactate selective impedimetric biosensor based on a LDH/ PyrOx bioselective membrane. Adapted from [72]. PyrOx: pyruvate oxidase; LDH: lactate dehydrogenase; BSA: bovine serum albumin; GA: glutaraldehyde; NAD^+^: nicotinamide adenine dinucleotide.

More recently, a novel lactate impedimetric bienzymetic biosensor based on lactate dehydrogenase and pyruvate oxidase was developed by Chan et al. (Figure 6) [72]. The biosensors exhibited a high operational and storage stability, and high selectivity, with the detection limits being 17 and 20 μM for the lactate dehydrogenase (LDH (EC 1.1.1.27)) layer and pyruvate oxidase (PyrOx (EC 1.2.3.3)) layer, respectively. The determination of l-lactate in complex matrices showed an applicability of the impedimetric enzyme-based biosensor for food quality analysis or clinical diagnosis. Table 2 is a brief summary of the types of analytes, immobilized enzymes, the immobilization method, and the limits of detection (LODs) of impedimetric enzyme biosensors.

### 2.2. Enzyme-Based Optical Biosensors

Enzyme-based biosensors using optical transducers have been evolved over nearly half a century. The optical transducers of enzyme-based biosensors measure changes in optical properties such as fluorescence intensity, light absorption, reflectance, chemiluminescence, evanescent wave, reflective index, and Raman scattering, resulting from the interaction of a biocatalyst with a target analyte. One of the earliest examples of an optical biosensor for clinical applications is a test strip for glucose in urine, commercialized in 1957 [80]. The working principle of the sensor utilized a cellulose pad coimmobilized with GOx and peroxidase in a cascade manner. Firstly, GOx catalyzes the oxidation of glucose to gluconic acid and hydrogen peroxide. The second immobilized peroxidase enzyme then catalyzes the reaction between the formed hydrogen peroxide and orthotolidine, to yield a deep blue-colored product. The change in blue color could be visually determined by the eye, and was used as a semiquantitative measurement of glucose concentration in urine.

#### 2.2.1. Absorbance/Reflectance Transitions

In a light absorbance-based enzyme biosensor, reactions occurring on the transducer surface lead to changes in the chemical environment, which could modify the light absorption properties of the biorecognition element at specific wavelengths. A single fiber or fiber bundle is commonly used to bring light to the analyte–catalyst transducer surface. Throughout the enzymatic reactions, the transmitted or reflected light is returned to the detector via fiber(s), and is measured as a signal to monitor the modifications induced by the recognition element, analyte, or the product of the enzymatic reactions. An absorbance-based biosensor for glucose quantification using two enzymes, GOx, and horseradish peroxidase (HRP (EC 1.11.1.7)) was fabricated by an enzyme entrapped in a polyacrylamide gel [81]. Upon exposure to glucose, the reaction between HRP and H_2_O_2_ yielded some intermediate species, which showed different absorption spectra compared to HRP (absorbance peak of 424 nm). The reasons for this phenomena are attributed to changes in the oxidation state of the HRP’s heme group during an oxidative reaction. This signal change then could be used for H_2_O_2_ determination, and the substrate involved in the previous reaction can also be quantified. The sensor was able to quantify the synthetic serum glucose concentration in fruit juices, ranging from 1.5 to 300 mM, with a long-term stability of at least six months. The authors also tested the sensor’s capability to work in whole blood (after dilution) [82]. The sensor displayed a long-term stability of over 30 months, and more than 200 measurements, with a response time in the range from 10 s to 5 min, and a dynamic range of up to 2 mM, by bubbling oxygen through the solution. An optical biosensor based on a metal–chelate nitrilotriacetic acid (NTA) affinity immobilization method was developed by using immobilized methyl parathion hydrolase (EC 3.1.8.1) [83]. The operating principle of the biosensor is based on the absorbance measurement of the yellow enzymatic product (p-nitrophenol), which shows absorption at a wavelength of 405 nm, resulting from the catalysis of colorless methyl parathion by methyl parathion hydrolase [84,85]. In a different work, an optical biosensor for the quantification of nitrite in water was developed by Rosa et al. [86]. It is based on the measurement of the optical reflectance of cytochrome cd1 nitrite reductase immobilized in controlled pore glass (CPG) beads. When nitrite reversibly binds to the reduced form and oxidizes the enzyme, spectroscopic changes were induced and measured as signals. The biosensor shows a sensitive response to increased concentrations of nitrite in solution, especially at 460 nm, with a corresponding detection limit of 0.93 µM.

#### 2.2.2. Fluorescence

Many enzyme-based optical biosensors have relied on the detection of a fluorescent signal from an enzymatic reaction in which fluorescence signal changes may be the result of the consumption of a fluorescent substance, fluorescent product formation, or a secondary fluorescent reporter signal change, due to product formation [87]. These signal changes correspond to the initial reaction rate from which analyte concentration could be determined via Michaelis–Menten equations. The first approach in developing this type of biosensor is based on the intrinsic fluorescent property of enzyme. All enzymes are fluorescent in the UV region of the spectrum, due to the presence of the three fluorescent amino acids—phenylalanine, tyrosine and tryptophan—in their structures [88]. A biosensor for this approach is based on the fluorescence quenching or fluorescence increase of the enzyme or co-enzyme molecules upon the formation of an enzyme–substrate complex. For example, Hussain et al. immobilized yeast hexokinase in a silica sol–gel, and observed up to 25% quenching of fluorescence at 330 nm on addition of glucose [89]. Yeast hexokinase enzyme has intrinsic fluorescence (ex~295 nm, em~330 nm), with each monomer subunit containing two lobes with a cleft in the middle. The fluorescence quenching effect is proposed to occur, due to changes in subunit molecule conformation; upon glucose binding to the enzyme active site, the two lobes in the cleft move closer together, which results in a quenching of fluorescence [90]. An in vivo fluorosensor was developed based on this scheme, by applying a glucose-permeable membrane on the enzyme layer [91], the sensor showed a linear range of glucose detection of up to 20 mM. The main drawback of using the fluorescence method of the intrinsic enzyme is that the excitation and emission wavelengths of the enzymes are limited within the UV region of the spectrum. This problem was then resolved by covalently bonding a fluorophore such as a coumarine derivative [92] or a fluorescein derivative [93] to the enzyme. The fluorescence changes during the enzymatic reaction were used for the batch determination of glucose [94], total cholesterol [95], and bilirubin [96]. As an example, Sanz et al. described an enzymatic fluorometric sensor for glucose determination in drinks [93]. The sensor was fabricated by immobilizing glucose oxidase, which is chemically modified with a fluorescein derivative (GOx-FS), in a polyacrylamide polymer as means of entrapment (photopolymerization). During the enzymatic reaction, changes in the fluorescence intensity of the GOx-FS were measured and correlated to the glucose concentration. Another approach employs the intrinsic fluorescence properties of enzymatic reaction cofactors such as nicotinamide adenine dinucleotide (phosphate) hydrogen (NAD(P)H) or flavine adenine dinucleotides (FADs). NADH has a strong absorbance at an absorption peak maximum of 340 nm, and a fluorescence emission peak at 450 nm, whereas NAD^+^ has no absorbance and emission capabilities at these wavelengths. The optical transducer can monitor either the absorption change at 340–360 nm, or the fluorescence emission change at 450 nm, and the signal is then correlated to the concentration of the target analytes. Biosensors built on this principle have been developed for the monitoring of glucose, cholesterol or l-amino acids [97], glucose-6-phosphate [98], sorbitol [99], glutamate [100], pyruvate or l-lactate. In a different approach, a fluorescent inhibitor is used to specifically bind to an enzyme in the presence of a specific cofactor. For example, Thompson and Jones employed this approach for sensing Zn^2+^, based on the specific binding of a dansylamide inhibitor to a Zn^2+^ cofactor [101]. Firstly, Zn^2+^ binds to the active site of carbonic anhydrase (EC 4.2.1.1); following that, dansylamyde binds to zinc. The binding results in an emission of a blue fluorescence only when zinc is present in the active site. Meanwhile, in the absence of zinc, dansylamyde does not bind to the enzyme active site, and thus exhibits weak fluorescence in the buffer. The approach was used to build a fiber-optics-based sensor for Zn^2+^ determination in a concentration range of 50 to 1000 nM.

#### 2.2.3. Luminescence

Luminescence is a phenomenon that occurs when an excited molecule emits light while returning to the ground state. The emitted light has a longer wavelength and a lower energy compared to the absorbed light [102]. In an enzymatic reaction, the presence of molecular oxygen would induce photoluminescence quenching of such excitable molecules by a radiationless deactivation process involving molecular interaction between oxygen and the fluorophore. Typical fluorophores (probes) include luminescent complexes of ruthenium [103], platinum [104], or palladium [105], which are strongly quenched by oxygen. Hence, the most widely used approach in luminescent enzyme-based biosensors is the measurement of oxygen consumption in fluorescence quenching by the means of luminescence intensity or lifetime measurement. One example of this type is a biosensor developed for glucose monitoring [106]. Sensitive optical coatings are formed from a commercial inorganic–organic hybrid polymer combined with a ruthenium complex and GOx. Bioluminescence, as a form of chemiluminescence, refers to the production and emission of light by living organisms such as fireflies and glowworms. In enzyme-based biosensors, luciferase enzyme is the most commonly employed enzyme for sensing based on bioluminescence. Gautier et al. [107] have investigated the use of luminescent enzyme systems linked to optical transducers for the determination of NADH, sorbitol, ethanol, and oxaloacetate at the nanomolar level. The multi-enzyme system was composed of a bacterial luminescence enzyme (bacterial luciferase (EC 1.14.14.3)) coimmobilized with other NAD(P)H-dependent enzymes such as sorbitol dehydrogenase (EC 1.1.1.14), alcohol dehydrogenase (EC 1.1.1.1), and malate dehydrogenase (EC 1.1.1.37) covalently attached on preactivated nylon membranes, which were attached to the end of a fiber-optic bundle and placed in a flow-through cell. The NADH formed by the reaction of the analyte with NAD^+^ in presence of the dehydrogenase enzyme was detected by using the bacterial luminescence fiber-optic sensor. In luminesensors, chemiluminescence is also applied to build enzyme-based biosensors. Chemiluminescence is a phenomenon resulted from the oxidation of certain substances, usually O_2_ or H_2_O_2_, to produce light without exciting illumination. The typical chemiluminescence reaction of luminol (5-amino-2,3-dihydro-I,4 phthalazinedione) with H_2_O_2_ formed during catalytic oxidation, in the presence of excess HRP, has been used to monitor various analytes such as glucose [108], phenolic compounds [109], glutamine [110], and hydrogen peroxide [111]. A chemiluminescence (CL) flow-through biosensor for glucose with eggshell membrane as an enzyme immobilization platform was developed by Li et al. [108]. The researchers developed a novel chemiluminescence flow-through biosensor for the detection of glucose by using HRP attached onto the eggshell membrane by chemical cross-linking with glutaraldehyde. The glucose was transformed to d-gluconic acid and hydrogen peroxide (H_2_O_2_) by GOx, and then CL emission occurred through the oxidation of luminol by H_2_O_2_ in response to HRP. The linear range and detection limit of the proposed biosensor were from 1 × 10^−6^ to 1 × 10^−4^ M, and 5 × 10^−7^ M, respectively. The biosensor based on CL displayed maintenance of good stability at 4 °C over a 5-month period, and was also expanded to measure glucose in human serum.

**Figure 7:**
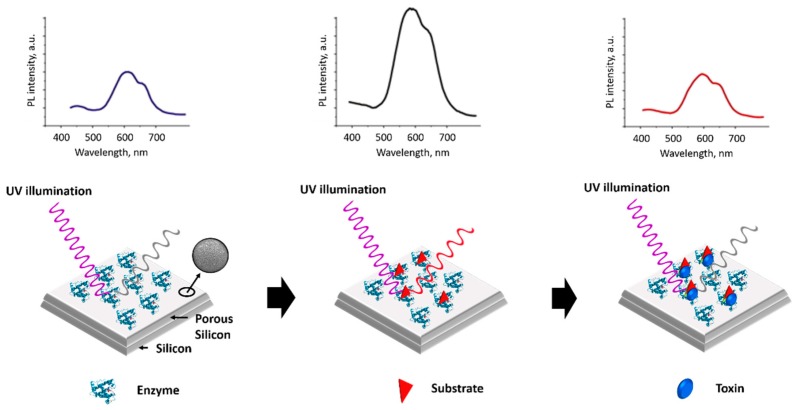
Schematic diagram of luminescent biosensors based on glucose oxidase or urease for the detection of glucose, urea, and heavy metals. Adapted from [112].

Recently, a porous silicon photoluminescence-based enzyme sensor for glucose detection was reported by Syshchyk et al. [112]. The luminescent biosensors for glucose, urea and heavy metals detection are schematized in Figure 7. Enzymatic reactions (i.e., urea–urease and glucose–glucose oxidase) were utilized for the direct determination of glucose and urea, by changing the optical properties of nanoporous silicon. The change in the photoluminescence of porous silicon occurred in response to urease or glucose oxidase. The photoluminescence intensity upshifts or downshifts by adding substrates to the sensor layer at various pHs. The heavy metal ions as enzyme inhibitors cause the restoration of photoluminescence quantum yield of the porous silicon through the interruption of the enzymatic reaction.

#### 2.2.4. SPR-Type Biosensors

**Figure 8:**
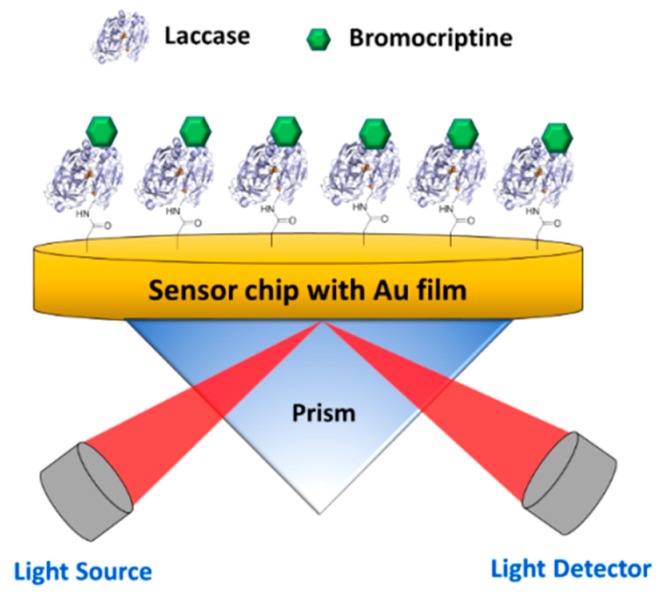
Schematic diagram of a novel enzyme based surface plasmon resonance (SPR)-biosensor for the detection of bromocriptine (BC). Laccase was used to design a SPR-based affinity biosensor for BC detection. Adapted from [115].

Another interesting approach for enzyme-based optical transducers is the use of the surface plasmon resonance (SPR) technique, which is based on the detection of changes in refractive index/light incident angles when biomolecules bind to the sensor surface. The sensor surface normally comprises glass substrate and a thin gold film. An incident polarized light is used to induce the SPR phenomenon, which occurs at specific angles of incidence, where a portion of the light energy couples through the gold film and creates a surface plasmon wave that is perpendicular to the incident surface. The binding of biomolecules to the sensor surface, which increases refractive index of solution near sensor surface (~300 nm), is attributed to changes in the momentum of the surface plasmons and their associated evanescent wave. As a consequence, the SPR phenomenon occurs at a new incident angle, which results in a SPR angle shift. This shift is directly proportional to the change in mass at the Au surface, and is used to monitor the association with and dissociation of biomolecules from the surface. A thorough review on SPR-based biosensor applications is found here [113]. SPR-based biosensors using immobilized enzymes have been developed for the studies of substrate–enzyme interactions [114], ligand–receptor interactions [115], and inhibitor screening [116]. Figure 8 shows a schematic representation of the biosensor constructed by immobilizing laccase (EC 1.10.3.2) onto a SPR surface for bromocriptine (BC) detection [115]. In that study, the biosensor showed high sensitivity with a linear range from 0.001 ng/mL to 1000 ng/mL, and a low detection limit of 0.001 ng/mL. It is thought that the binding of BC to the serum albumin enhanced the SPR signal, resulting in acceptable refractive index changes, despite its low molecular weight.

Another novel SPR-based biosensor was developed for small molecule detection by Miyazaki et al. [117]. The sensor makes use of the mediator-type enzyme microperoxidase-11 (MP11) and poly(ethylene imine) (PEI) for the construction of layer-by-layer films, as in Au/PEI/MP11/PEI/Gox and Au/PEI/MP11/PEI/Uox for glucose or ureic acid detection with GOx or uricase (EC 1.7.3.3), deposited on the top layer of the films, respectively. The fabricated SPR sensor was able to detect glucose or uric acid with limits of detection of 3.4 and 0.27 μmol L^−1^, respectively. The main advantage of this sensor is the use of the mediator-type enzyme (MP11), as it can be applied to any other sensing system where hydrogen peroxide is generated in an enzymatic reaction. Other SPR-based enzyme biosensors have been reported for the detection of cholesterol [118] and hydrogen peroxide [119]. A study of substrate-induced conformational changes using a SPR-based biosensor was performed by Geitmann et al. [120]. In that study, the interaction between human cytomegalovirus (HCMV) protease and a peptide substrate was studied by using SPR. The HCMV protease was chemically cross-linked to the sensor surface to limit the structural flexibility of the enzyme. As a result, enzyme activity was inactivated. However, the sensor gram analysis and the kinetic constants calculation showed that upon flowing the peptide substrate onto the chip surface, the enzyme–substrate interaction restored enzyme activity. It is therefore supposed that the HCMV protease undergoes a conformational alteration during the hydrolysis of a polypeptide substrate, because this enzyme requires structural flexibility to be active.

### 2.3. Enzyme-Based Thermistors

Almost all enzymatically catalyzed reactions are exothermic or generate heat, which may be used as a basis for measuring the rate of reaction and the analyte concentration (Table 3). The total generated heat (Q) is proportional to the molar enthalpy change (∆H), and to the total number of moles of product molecules (nP). A thermistor is a type of resistor resistance that is dependent on temperature, and it measures changes in temperature by ∆H in the form of electrical signals, such as resistance. Thermistor-based calorimeters, popularly known as enzyme thermistors (ET), use thermistors to measure electrical changes due to changes in temperature following a biocatalytic reaction, and this system is especially exploited for quantification purposes. The operating principle of an ET is simple. The enzyme is immobilized in a packed bed column within a constant temperature environment. The enzymatic reaction takes place when substrate enters the bed. As a result, the substrate is converted to a product, together with heat release. The difference in the temperature between the substrate and product solution is measured by two thermistors placed at the entrance and exit of the column. Highly sensitive thermistors are used, so that even a small change in the temperature can be detected by thermal biosensors. The used substrate amount is quantified based on the amount of heat that is liberated, in a directly proportional manner. The enzyme thermistor is a direct method that is insensitive towards electrical or optical interferences, and it does not usually require additional reagents. In addition, it offers high enzyme loading capabilities, excellent operational stability, and long storage, together with the capability to be combined with a flow injection analysis (FIA) system, which allows high reproducibility, high throughput, and the possibility for continuous analysis [121]. The main drawbacks of enzyme thermistor is non-specificity, because thermal signal is dependent only on the underlying reaction.

**Figure 9:**
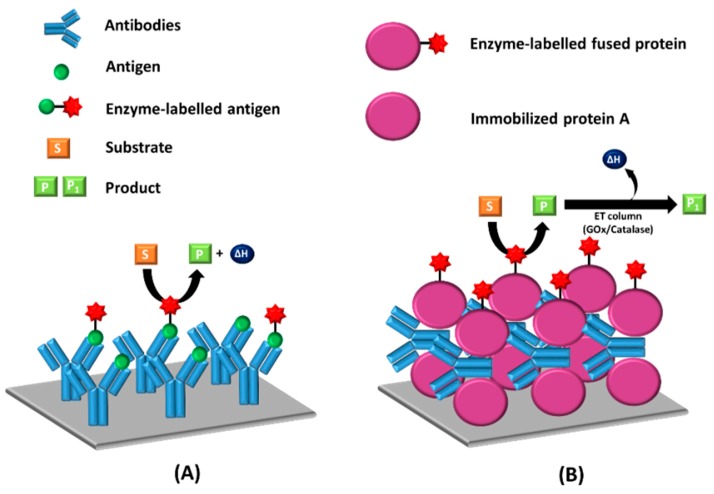
Schematic representation of the thermometric enzyme-linked immunosorbent assay (TELISA) method. (**A**) Direct competitive TELISA; (**B**) sandwich TELISA. Adapted from [124].

The thermometric enzyme-linked immunosorbent assay (TELISA), for the assay of endogenous and exogenous compounds in biological fluids has been developed by Mattiasson et al. [122]. The TELISA is based on an enzyme-linked immunosorbent assay (ELISA), but it utilizes heat produced from the enzyme label that is measured by using an enzyme thermistor. There are two types of TELISA (i.e., direct competitive immunoassay [122] and the sandwich immunoassay [123]). The amount of the enzyme that bound to the antibodies is measured with the ET unit by adding the cognate substrate. In a direct competitive format of TELISA, the produced signal is reversibly proportional to the concentration of the analyte. A simple TELISA based on the sandwich format, which is a calorimetric immunoassay using an ET, was established by Scheper et al. [123]. The authors developed a sandwich assay with protein A immobilized onto a solid support for capturing antibodies. In that study, protein A-fused β-galactosidase obtained from a recombinant *E. coli* was used in labeling and reaction for detection. The signal produced in sandwich TELISA is directly proportional to the concentration of antibodies that are present in a sample. Figure 9 shows the schematic illustration of the TELISA method for both direct competitive (A) and sandwich (B) formats.

### 2.4. Enzyme-Based Piezoelectric Biosensors

The most common type of piezoelectric biosensor is quartz crystal microbalance (QCM), which is able to determine nanograms of material. The sensor consists of a thin wafer of quartz-sensing crystal plated with metallic electrodes on either sides of the crystal by means of vapor deposition. When an AC voltage is applied across the crystal, the induced piezolectric effect causes it to oscillate at its resonant frequency. Any adsorption of molecules to the surface of the oscillating crystal will cause its frequency to decrease. By measuring this frequency change, the amount of mass per unit area deposited on the surface can be determined with great precision (down to a few billionths of a gram). In an enzyme-based QCM biosensor, the resonance frequency decreases upon the adsorption of the enzymatic product onto the sensor surface. The frequency change (ΔF) is proportional to the mass (Δm) of the adsorbed molecules per unit area. A QCM-based piezoelectric biosensor was developed for urea detection by immobilizing urease onto nanoporous alumina membranes by the means of physical adsorption and cross-linking [125]. The relative enzyme activity was estimated by measuring the frequency response of the sensor in solutions of urea concentration, ranging from 0.2 µM to 12 mM. The experimental results showed a good linearity for urea concentration, over a range from 0.5 µM to 3 mM (with the linear regression equation was ∆F (Hz) = −17.85 − 164.8 [urea, mM], R = 0.9996, n = 8). A detection limit of 0.2 µM (S/N = 2) for urea was obtained, and the sensor showed good long-term storage stability (76% of the enzymatic activity retained over 30 days).

**Figure 10:**
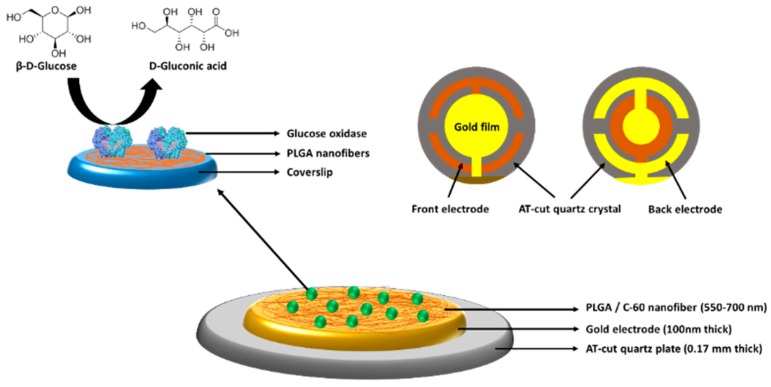
Schematic representation of the electrospun nanofibrous PLGA/Fullerene-C60-modified QCM for the real-time monitoring of gluconic acid. Adapted from [126].

A Poly(lactic-co-glycolic acid) (PLGA)/C60-QCM sensor using immobilized GOx for the real-time determination of gluconic acid was developed by Seker et al. [126]. As shown in Figure 10, the quartz crystals were coated with a 550–700 nm-thick layer of nanofibers comprised of PLGA and fullerene-C60 by electrospinning. Then, GOx was immobilized on the PLGA nanofibers, which were electrospun on coverslip surfaces. During the enzyme catalytic reaction, gluconic acid—the oxidation product of β-d-glucose—was induced and precipitated onto the crystal surface, resulting in a resonance frequency decrease. As a result, a LOD in the range of 1.4–14.0 mM for gluconic acid at room temperature was obtained. An enzyme-based piezoelectric biosensor for detecting organophosphorus and carbamate pesticides was described in Abad et al.’s work [127]. The authors directly immobilized AChE onto QCM gold electrodes by covalent bonding between the enzyme and the gold surface. The AChE-modified QCM sensor showed detection limits of 5.0 × 10^−8^ and 1.0 × 10^−7^ M for paroxon and carbaryl, respectively. Piezoelectric sensors have been attractive, due to their simplicity, real-time measurement, high sensitivity, and cost-effectiveness. However, the major drawbacks of these devices are the interference from atmospheric humidity, and the difficulty in applying for the determination of the material in solution. The common types of analytes, immobilized enzymes, immobilization methods, and the detection range/LOD of enzyme-based QCM sensors are outlined in Table 4.

## 3. Approaches in Improving Enzyme Usage in Biosensors

In this section, approaches in improving the use of enzyme for biosensors will be discussed with regards of enzyme modification by genetic and chemical approaches, and multi-enzyme systems used in biosensors.

### 3.1. Biological Modification

Recent advances in genetic engineering and molecular biology has allowed for the production of high efficient and high specific recombinant enzymes, which are applied for the improvement of biosensor performance. The key technique is to increase the affinity of enzyme–substrate by facilitating substrate accessibility to the enzyme active site. In enzyme engineering, this purpose can be obtained using techniques such as site-directed mutagenesis or protein fusion [133].

#### 3.1.1. Site-Directed Mutagenesis

Site-directed mutagenesis (SDM) is a method for creating specific and targeted changes in double stranded plasmid DNA [134]. Site-directed mutagenesis is employed to alter the amino acid sequence of a given enzyme molecule by carefully selecting and precisely mutating the cloned gene encoding the corresponding amino acid on the enzyme molecule. This technique is usefully applied to the study of protein function, the identification of enzymatic active sites, and the design of novel proteins. With this technique, it is possible to exchange, remove, or add a single amino acid or other genetic tags into the sequence of an enzyme to achieve different chemical properties.

##### Enzyme Amino Acid Substitution

Each of the twenty natural amino acids has a unique side chain that has different properties with regard to size, shape, and polarity, and enzyme function is closely related to the amino acid sequence. For that reason, the substitution of any natural amino acid (AA) with another one may lead to major changes in enzyme structure and function. As an example, genetically modified AchEs have been widely exploited in pesticide biosensors, owing to their inhibition effects towards pesticides [135]. Genetically engineered *Drosophila melanogaster* AchEs were made by replacing glutamic acid 69 (Glu69), located at the enzyme active site gorge with bulky side chains amino acids such as tryptophan (Trp) or tyrosine (Tyr). The engineered enzyme has been demonstrated to greatly increase the inhibition constant (K_i_) for dichlorvos by 300 folds [136]. This effect is attributed to alterations on the residues of the active region [137] which favor the interactions of the insecticide towards the buried active site. Furthermore, a more sensitive AchE enzyme (with a 20,000-fold increase in K_i_) was obtained by replacing tryptophan 71 (Tyr71) with aspartic acid (Asp) in addition to the Glu69 substitution. In another work, mutation is also made to pyrroloquinoline quinone glucose dehydrogenase (PQQ-GDH (EC 1.1.5.2)) by replacing the His residue at position 775 with Asp or Asn, to produce enzymes with more than 25-fold increases in the Michaelis constant (K_m_) value towards glucose [138]. The biosensor based on the coimmobilization of the two mutated PQQ-GDH (1:1 ratio) showed high specificity and a wider dynamic range for glucose detection (3–70 mM) compared to that of the wild enzyme-based biosensor.

##### Enzyme Amino Acid Removal

Amino acids that are not essential for the enzyme functionality could be eliminated to facilitate the electron transfer, thus making the active site more accessible. For example, microperoxidase-11 (MP11), an undecapeptide obtained by the enzymatic cleavage of cytochrome c from HRP while retaining the heme-c group, still exhibits peroxidase activity. The minimized enzyme has been successfully used as an efficient biorecognition molecule in peroxide sensors [139], and as a bioelectrocatalytic label in enzyme sensors [140].

##### Non-Natural Amino Acid Incorporation

Site-directed mutagenesis using natural amino acids has proven to be useful in enzyme engineering; however, it has some limitations, due to restrictions in the size and shape of the natural amino acid side chains. Therefore, site-directed mutagenesis using non-natural amino acids has been explored for the expression of recombinant proteins containing amino acids with novel side chains, including fluorophores, post-translational modifications, metal ion chelators, uniquely reactive functional groups, or photocross-linking moieties, etc. The technique, which allows for control over the chemical structures of recombinantly expressed proteins, hence could provide novel target proteins with new functionalities that cannot be obtained by using natural amino acids. Intrinsic enzyme properties such as fluorescence and catalytic activity could also be improved by using the site-directed mutagenesis technique [141]. Non-natural amino acids can be incorporated into enzyme molecules via chemical synthesis, or into vitro cell-free protein translation systems. Also, cell-based protein expression systems could be employed for large-scale manufacturing purposes [142]. For example, the bacterial enzyme phosphotriesterase (EC 3.1.8.1), which catalyzes the hydrolysis of pesticide paraoxon, was modified for turnover rate improvement by the incorporation of unnatural amino acids [143], where the tyrosine at position 309 was substituted for unnatural 7-methyl- and 7-hydroxycoumarinyl amino acids (Mco and Hco). Kinetic analysis revealed that the product release of substrate turnover increased by 8–11 times by the means of a single mutation that is rationally designed by using unnatural amino acids. The method is far more facile than the native activity improvement method, by screening hundreds of thousands of mutants with natural amino acids. In another work, natural aldolases was engineered by the means of noncanonical amino acid incorporation, to produce enzymes with higher substrate specificities. By modifying position 190 of the *S. aureus N*-acetylneuraminic acid lyase (NAL (EC 4.1.3.3)) to a 2,3-dihydroxypropyl cysteine, the enzyme activity for the reaction of the aldol reaction of erythrose with pyruvate to form DHA is significantly increased by approximately 30-fold, which is unattainable when using any of 20 tested natural amino acids. The reasons behind this boost are that when the functional and noncanonical amino acid is accurately inserted between Asp141 and Glu192, the enzyme active site volume is reduced, and undergoes a remodeling that thus helps better stabilize the transition state of the DHA-producing reaction.

##### Enzymatic Addition of a Genetic Tag

Another approach in site-directed mutagenesis is genetic tag modification, which could reduce steric hindrance during the affinity-based immobilization of tagged enzymes. The approach allows for the production of tagged enzymes by attaching an affinity tag such as histidine (His), cysteine (Cys), or mannose-binding protein to amino or carboxyl terminals that are distant from the enzyme active site. Enzymes have complex structures and activities. Therefore, in an ideal immobilization approach, enzymes must be immobilized in their native form to maintain their functional conformation, and to maximize their substrate capture potential. In addition, the non-specific adsorption of enzymes onto a solid surface can lead to the possibility of background problems. The affinity interaction between transition metal ions (Au^+^, Ni ^2+^, Co^2+^, Cu^2+^, Zn^2+^, Mn^2+^ and Fe^3+^), and electron-donating groups like His and Cys residues, are utilized for the controllable immobilization of the enzyme. For example, the native protein or enzyme is added with a metal binding site [144], a His residue [145] or a Cys residue [146], to obtain a uniform orientation for the enzyme immobilization on surface. Also, ferrocene derivatives could be linked to an enzyme (GOx) which is genetically modified with a poly-l-lysine, to facilitate electron transfer [147].

#### 3.1.2. Fusion Protein Technology

Fusion protein technology is a biotechnological tool that is used to engineer protein molecules by joining two or more genes that originally coded for two different proteins. The translation of this fusion gene results in a so-called fusion protein with functional properties that are derived from each of the original proteins. The advantages of using fusion proteins over coimmobilized enzymes are that the resulting protein molecules have fixed molecular ratios of individually immobilized enzymes, thus ensuring the desired activity of the enzyme membrane. In addition to improved stability and sensitivity, a convenient “tag” could be employed as a fusion partner for ease of detection. A bifunctional fusion enzyme system constructed for a maltose biosensor was developed by gene splicing technique [148]. The fusion enzyme system of glucoamylase (GA, (E.C 3.2.1.3)) and GOx, by fusing the complementary DNA (cDNA) fragment of *Aspergillus niger* glucoamylase to the 3’ end of the *A. niger* GOx gene with the insertion of a flexible linker peptide (-(Ser-Gly)5-) coding sequence was reported by Chen et al. [149]. The obtained fusion enzyme GOx-(Ser-Gly)5-GA (GLG), after methanol induction, had a molecular weight of 430 kD and showed the typical kinetic properties of both GA and GOx, from kinetic analysis data. After being covalently attached onto an aminosilanized glass slide through glutaraldehyde, data showed that GLG presents a much higher sequential catalytic efficiency than that of the GA and GOx mixture (GA/GOx). A linear response range of up to 40 mM for maltose detection was obtained for the GLG electrode. In another work, a fusion of GOx and a poly-lysine chain was constructed by means of gene splicing and used to build a glucose sensor with improved signal levels, response ranges and lifetime. The poly-lysine chain was added to the C-terminal of GOx through a peptide linker, in order to anchor more electron transfer mediator (ferrocenecarboxylic acid) to GOx, thus improving the sensitivity and stability of glucose biosensors. The modified GOx showed similar Km and Kcat to those of the wild type enzyme. Meanwhile, in comparison with commercial GOx and wild type GOx, (*A. niger*), higher enzyme activity remained after the interaction of the modified GOx with an electron transfer mediator (90.01%, compared to 22.43% and 22.17%). Three types of GOx: the modified, wild type, and commercial type were coated on screen-printed electrodes and tested for glucose sensitivity comparison. The experimental results showed that the modified GOx coated electrode gave the largest signal responses among the three tested electrodes. In addition, a linear detection range was extended to 45 mM for the modified GOx-based biosensor compared to 20 mM for the wild type and the commercial GOx-based biosensors.

### 3.2. Chemical Modification

Chemical modification is an effective method that is used to change the properties of enzyme key residues or the overall surface [150]. Although it has generally been displaced by genetic modification, the chemical modification of enzymes remains useful for stabilizing proteins, owing to some distinct advantages [151]. For example, there are no limits to the range of chemical groups to be introduced to the enzyme structure, using chemical modification [152]. The method is rapid (compared with genetic modification), and does not require a deep knowledge of the protein structure. In addition, chemical modification is performed on the correctly folded enzyme; meanwhile, in genetic modifications, changes in the amino acid sequence could hinder the correct folding of the protein. However, unlike genetic modification, chemical modification needs to be performed every time the enzyme is prepared, while in genetic manipulation, the mutated gene will always produce the enzyme with desired changes. Moreover, site-directed modification using chemical method is rather difficult, compared with site-direct mutagenesis by using genetic modification.

#### 3.2.1. Site-Specific Chemical Modification

Chemical modification will not be generally site-directed, except when using specific chemistries that are directed to unique groups in the protein structure such as Diels–Alder cycloaddition [153], thiol exchange [154], reaction with terminal amino groups [155], etc. For example, the tetrazine–trans-cyclooctene Diels–Alder cycloaddition is used as a highly efficient fluorescence labeling method for cell-surface proteins and for the labeling of intracellular proteins with sufficiently bioorthogonal chemistry. Cellular proteins of interest were labeled with useful fluorophores, such as tetramethylrhodamine and Alexa 647, with a level of specificity that was comparable to that obtained with direct fluorophore ligation by the PRIME (probe incorporation mediated by enzymes) method [153]. Among 20 natural amino acids, cysteine is the most reactive amino acid residue for selective modification, due to the relatively high nucleophilicity of the sulfhydryl groups (thiols), and it therefore can be modified by a large number of reagents. In proteins that contain only cysteine residues (natural or by site-directed mutagenesis), selective protein/enzyme conjugation (labeling) could be made possible via thiol exchange [154]. Site-specific chemical modifications methods have been developed for N-terminal amino acids (amino acids that start with a free amine group). Tryptophan residues located in the N-terminal can be modified through the stereoselective Pictet–Spengler reaction with aldehydes [156]. N-terminal cysteines could react with thioesters [157] or readily form thiazolidines in the presence of aldehydes [158]. N-terminal serine and threonine residues can be selectively oxidized by sodium periodate to form glyoxylamides [159], which can then be modified with hydrazide or aminooxy reagents.

#### 3.2.2. Nonspecific Modification of the Enzyme Surface

Nonspecific chemical modification by a massive modification of abundant external residues (i.e., Lys or Asp and Glu) on enzyme may change the overall properties of the enzyme surface [160], which may greatly alter its properties. For example, by chemical amidation using ethylenediamine, carboxylate groups in Asp and Glu residues in several immobilized lipases have been modified to modulate lipase activity. Significant increases in enzyme activity were observed in the case of *Candida antarctica* B and *Thermomyces lanuginose* lipases, while in case of immobilized *Pseudomonas fluorescens* lipase, the result was opposite [161]. Chemical modification of lysine residues in a bacterial α-amylases (EC 3.2.1.1) from *Bacillus amyloliquefaciens* (BAA) using citraconic anhydride brought about a dramatic enhancement of the thermal stability of BAA (at 80 °C) [162]. A shift in the optimum operating pH of enzyme from 7 to an alkaline pH of 9 was induced by the nonspecific modification of amino groups of papain with dicarboxylic anhydrides of citraconin, maleic, phthalic, and succinic acids [163]. The reason for this difference by pH change is because of the change in the surface charge distribution. In addition, experimental data showed a slight decrease in enzyme activity as the result of the modification. Such optimum pH changes may be useful when performing reactions that favor alkaline media or the enhancement of particular substrate solubility. In general, it is not easy to anticipate the effects of a chemical modification on enzyme properties, because it is usually dependent on certain experimental conditions such as pH and temperature [158]. In certain cases, chemical modifications may produce an improvement in the enzyme properties but in other cases, it may cause a decrease in the enzyme reactivity and selectivity.

#### 3.3.3. Chemical Cross-Linking

**Figure 11:**
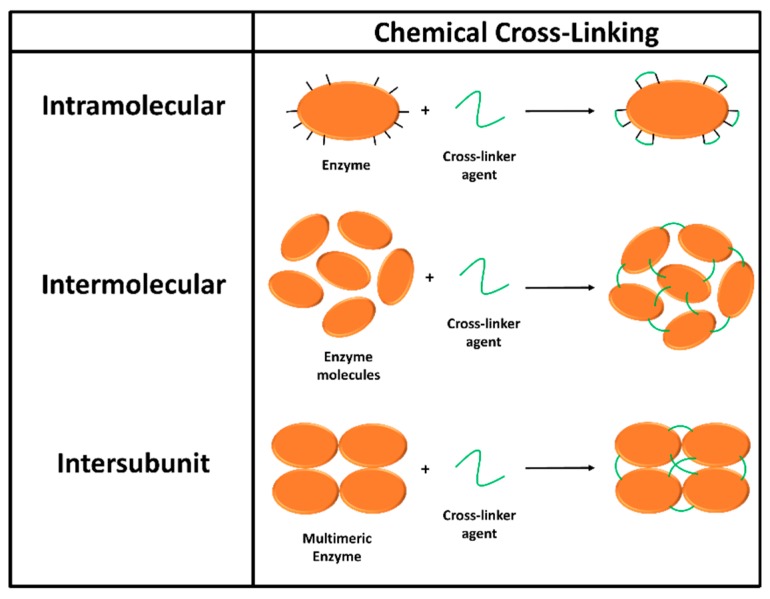
Different types of chemical cross-linking and modification of enzymes. Adapted from [164].

The establishment of chemical cross-linking between several different groups of the enzyme surface using reagents that contain two or more reactive functional groups can be used to form intramolecular, intermolecular, or intersubunit cross-links between amino acid residues of proteins (Figure 11) [3,164]. Among a variety of reagents utilized for this purpose, glutaraldehyde is generally used as the cross-linking agent of favor, because it is inexpensive and readily available in large quantities. Glutaraldehyde has been used for decades in protein cross-linking [165].

Intramolecular cross-linking method uses a cross-linker agent to link involved functional groups onto a protein/enzyme surface, so that the relative distance between the groups cannot become larger than the size of the agent. The method is therefore able to restrict any conformational change of the enzyme that is induced by inactivating agents (i.e., heat, solvent, and chaotropic reagents). As a result, the overall rigidity and stability of the enzyme would be increased. However, the cross-linker agent is required to have a length that fits to the distance between involved groups [166]. Intramolecular cross-linking of enzymes has been found to contribute to the enhancement of enzyme conformational stability [167], and the stabilization of the enzyme against thermal inactivation [168]. In the case of intersubunit cross-linking, the covalent bridges created by cross-linker need to be located in the contact area between enzyme subunits to avoid subunit dissociation, efficiently improving enzyme stability in that way [169]. Intermolecular cross-linking of enzymes of interest is also an efficient method in stabilizing oligomeric enzymes. It is because the formation of covalent bonds between molecules of the enzyme and the reagent would result in a three-dimensional, cross-linked network that could help preventing the dissociation of their sub-units [170]. There are two approaches in intermolecular cross linking, which are the uses of cross-linking enzyme aggregate (CLEA) and cross-linking enzyme crystal (CLEC). Both methods require the use of a cross-linking agent such as glutaraldehyde, to cross-link enzyme molecules via the reactions of the free amino groups of lysine residues on the reactive site of neighboring enzyme molecules. For example, CLECs of cyclodextrin glucanotransferase (CGTase (EC 2.4.1.19)) were tested for thermal stability at an elevated temperature, in organic solvents, and in the presence of the enzyme inactivation surfactant [171]. The test results showed that the soluble CGTase exhibited no activity at 80 °C whereas CGTase-CLECs retain 48% of their original activity at that temperature. In the presence of proteases, or in organic solvents, CGTase-CLECs also maintained their activity, and thus they have a great potential for biosensor applications. CLEAs, which are an improved versions of CLEC, proved to be significantly more stable to heat denaturation, organic solvents, and proteolysis than the corresponding soluble enzyme [172]. As an example, the precipitation of lipases from *Thermomyces lanuginosus* and *Rhizomucor miehei* with (NH_4_)_2_SO_4_ in the presence of sodium dodecyl sulfate (SDS), followed by cross-linking with glutaraldehyde, resulted in CLEAs with three-fold and two-fold increases in the hydrolytic activity compared with native enzymes, respectively [173].

#### 3.3.4. Use of Polymers

The modification of proteins with polymers is one of the most widespread approaches for creating hybrid structures with improved characters, owing to some unique advantages [174]. First, the incorporation of polymers into enzymes could improves enzyme stability, solubility, and biocompatibility [175]. Second, multi-functional polymers that contain many reactive groups can serve as cross-linking agents for multiple intramolecular or intersubunit cross-linkings of enzymes, which result in large complexes with increased sensitivities or activities in detecting target analytes. A polymer probably contributes to improving the stability, solubility, and biocompatibility of an enzyme, according to the following reasons: (1) The confinement of enzyme movements by the presence of a bulky polymer near the enzyme surface; (2) The prevention of enzyme interactions with interfaces by effectively masking the intrinsic character of the surface; (3) The changes of the enzyme environment; (4) Reduced proteolysis or reduced oxidation by different reagents [176]. However, the use of polymer could limit the rigidification of the enzyme, owning to the flexible structure of the polymers, and the existence of loops between polymeric bonds.

**Figure 12:**
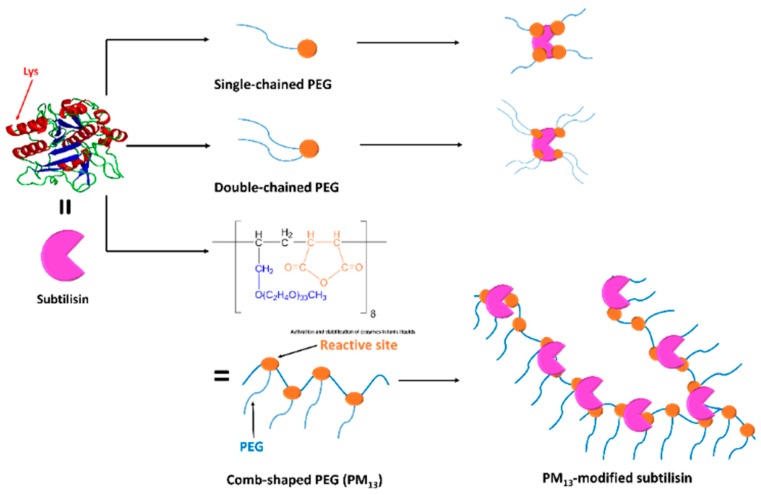
Schematic diagram of the modification of subtilisin with poly(ethylene glycol) (PEG) molecules. Adapted from [177].

Two of the most generally used polymers in the chemical modification of enzyme are dextran and polyethylene glycol (PEG). Subtilisin (EC 3.4.21.62) as a model enzyme was covalently modified with PEG in Nakashima et al.’s study [177]. The researchers evaluated the catalytic behavior and the solubilization of subtilisin in ionic liquids with chemical modification with comb-shaped poly-(ethylene glycol) (PM_13_) (Figure 12). The PM_13_-modified subtilisin was solubilized in ionic liquids, and exhibited a remarkably high activity and good stability in the solutions. Chemical modification of HRP with poly(ethylene) glycol (PEG) has been reported to improve the enzyme activity in toluene by up to 16-fold, and result in a significant increase of enzyme solubility and stability in some organic media [178]. This is due to the ability of PEG to trap water on enzyme surface; thus it could preserve the three-dimensional structure of the enzyme in a catalytically active form [179]. Similarly, the covalent conjugation of GOx with 75 kDa dextran (1:5 molar ratio of GOx/dextran) yielded an enzyme with a high thermal resistance and good stability in a wide pH range (pH 4.0–7.0) at high temperatures (80 °C) [180].

### 3.3. Multi-Enzyme Systems

Multi-enzyme system refers to the use of two or more enzymes coimmobilized onto a sensor transducer surface to enhance biosensor performance [181]. The working principle of this multi-enzyme system is based on enzyme cascade reactions, which are comprised of several consecutive biocatalytic steps. In each enzyme-catalyzed step, an unstable intermediate is formed, and this intermediate then spontaneously undergoes further reactions before forming the ultimately stable product. The whole reaction process is sometime called enzyme-initiated domino reactions. An enzyme cascade reactions system offers advantages such as no requirement for intermittent product isolation, conservation of cost and reagent, high reversibility, and low inhibition. However, a hindrance to the use of the system is the availability of suitable enzymes which have similar pH, temperature profiles, and relative similarity in specific activities and stabilities. The use of enzyme cascade reactions has been thoroughly reviewed in the literature [182] and briefly covered in a few selected examples below. An electrochemical multi-enzymatic biosensor was used for the determination of free cholesterol [183] using a bienzyme system composed of HRP and cholesterol oxidase (EC 1.1.3.6). This was simultaneously immobilized by physical entrapment into a polymeric film, on the surface of a graphite electrode. A novel bi-enzyme modified-amperometric sensor for the detection of methyl salicylate released by pathogen-infected plants was developed by Fang et al. [184]. The measurement of the bi-enzyme biosensor, in which alcohol oxidase (EC 1.1.3.13) and HRP enzymes were immobilized on a carbon nanotube matrix by a molecular tethering method, was performed by cyclic voltammetry and constant potential amperometry. The sensitivities determined by each method were 112.37 and 282.82 μA·cm^−2^·mM^−1^ respectively, and the each detection limit was 22.95 and 0.98 μM.

**Figure 13:**
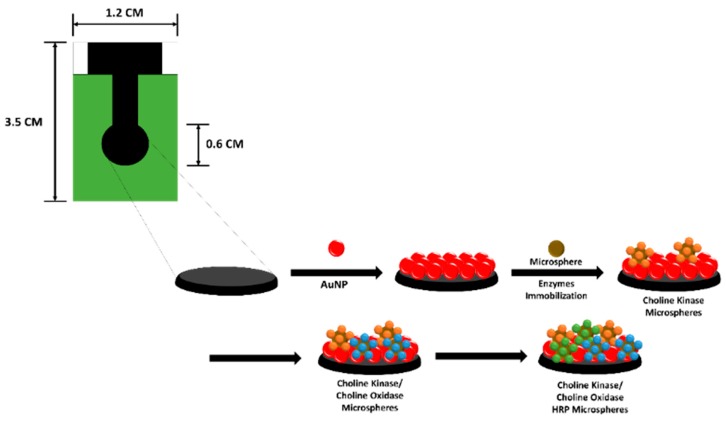
Schematic conception of a fabrication procedure for an amperometric biosensor to identify sPLA2-IIA as an indication of sepsis conditions. A tri-enzyme system consisting of choline kinase/choline oxidase/horseradish peroxidase was utilized to determine the target analytes by using cascading enzymatic reactions. These enzymes were immobilized onto acrylic microspheres functionalized with succinimide groups, which formed a composite with gold nanoparticles (AuNPs) to improve the electrode conductivity. The composite was deposited onto screen-printed electrodes (SPEs). Adapted from [185].

A luminescent biosensor based on tri-enzymes such as alcohol dehydrogenase, sorbitol dehydrogenase, and malate dehydrogenase was developed for measuring ethanol, sorbitol, and oxaloacetate [107]. Recently, Mansor et al. suggested an interesting tri-enzyme system composed of choline kinase/choline oxidase/HRP for the rapid and specific detection of secretory phospholipase Group 2-IIA, a biomarker for bacterial sepsis infection [185]. The amperometric sensor surface was modified with choline kinase/choline oxidase/HRP-conjugated acrylic bead/gold nanoparticle composite attached onto a carbon-paste electrode (Figure 13). The linear range of detection and the detection limit of the sensor were 0.01–100 ng/mL and 5 × 10^−3^ ng/mL, respectively. Another amperometric biosensor based on a tri-enzyme for the lysine determination as an index of the nutritional quality of pharmaceutical formulations and food samples was developed by Bóka et al. [186]. In their study, tri-enzymes including lysinedecarboxylase (EC 4.1.1.18) from *Bacterium cadaveris*, diamine oxidase (EC 1.4.3.6) from *Pisum sativum*, and HRP, were co-immobilized on the surface of a graphite electrode with an osmium redox polymer. The lysine sensor provided a linear detection range from 0.005 to 0.500 mM, and it showed selective determination of lysine in pharmaceutical products and food stuffs. Four enzymes (lactase (L), glucose oxidase (G), mutarotase (M) and galactose oxidase (Ga)) were appropriately combined to form uni-(Ga), di-(LG, LGa), tri-(LMG, LGGa), and tetra-(LMGGa) enzyme systems [187]. Six different lactose electrodes were formed, based on combinations of the four enzymes, and lactose was determined amperometrically by monitoring the hydrogen peroxide produced. Among the six electrodes, the tri-enzyme electrode, LMG, was dominant in terms of lactose response, with linear range of 3 × 10^−6^–2 × 10^−3^ M, even though the tri-enzyme electrode, LGGa, and the tetra-enzyme electrode, LMGGa, showed a two-fold increase in sensitivity over LMG. It is noteworthy that the MG electrode-based biosensor exhibited good storage stability for eight months at 4 °C, considering that the enhancement of long-term stability and the activity of immobilized enzyme membranes is important for all practical purposes of enzyme-based biosensors.

## 4. Conclusions

In recent decades, enzyme-based biosensing has proven to be a valuable technique for the qualitative and quantitative analysis of a variety of target analytes in biomedicine, environmental, food quality control, agricultural, and pharmaceutical industry. In comparison with conventional analytical methodologies, enzyme-based biosensors offer significant benefits, such as miniaturization, real-time diagnosis capability, high sensitivity and specificity, minimum sample preparation, and high-throughput, bedside clinical testing and portability. A number of developed enzyme biosensors have been already commercialized and used in health care management (i.e., home blood glucose monitoring, portable clinical analyzers, etc.). However, the most challenging disadvantages of the enzyme-based biosensor for in vivo analysis is a reduced signal response and selectivity, due to the presence of fouling agents and interference caused by chemicals present in the sample matrix. Among the efforts to address the problems, enzyme engineering and hybrid biomaterial incorporation has been used to promote enzyme stability and to minimize endogenous interference; the third-generation biosensor development are especially focused upon, as they offer interference-free, reagent-less and label-free detection of the analyte. The present developments trends of enzymatic biosensors are directed towards device miniaturization, multiplexed detection, and applicable expansion to bedside patient and home testing devices such as paper-based test kits, lab-on-a-chip and biochip sensing devices, which require minimum sample pretreatment, and low reagent and power requirements.

## Figures and Tables

**Table 1:** Summary of substrates, immobilized enzymes, and immobilization methods of conductometric enzyme biosensors.

Substrates	Immobilized Enzymes	Immobilization Methods	Ref.
Urea	Urease	Cross-linking (GTA ^(4)^)	[55]
Creatinine	Creatinine deiminase	Adsorption	[56]
l-asparagine	l-asparaginase	Cross-linking (GTA)	[57]
Glucose	GOx ^(1)^	Covalent bonding (EDC ^(5)^-NHS ^(6)^)	[58]
Lactose	GOx, β-galactosidase	Cross-linking (GTA)	[59]
Hydrogen peroxide	Peroxidase	Cross-linking (GTA)	[60]
d-amino acids	d-amino acid oxidase	Cross-linking (GTA) Covalent bonding (Hydrazine)	[52]
Organophosphates	AChE ^(2)^, BChE ^(3)^	Cross-linking (GTA)	[61,62]
Acetylcholine	AChE	Cross-linking (GTA)	[63,64]
Butyrylcholine	BChE	Cross-linking (GTA)	[63,64]
Heavy metal ions	Urease	Cross-linking (GTA)	[65]
Uric acid	Uricase	–	[66]
Formaldehyde	Alcohol oxidase	Cross-linking (GTA)	[67]
Triazine herbicides	Tyrosinase	Cross-linking (GTA)	[68]
Carbamate pesticides	AChE	Cross-linking (GTA)	[69]

^(1)^ GOx, Glucose oxidase; ^(2)^ AChE, Acetylcholinesterase; ^(3)^ BChE, Butyrylcholinesterase; ^(4)^ GTA, Glutaraldehyde; ^(5)^ EDC, 1-Ethyl-3-(3-dimethylaminopropyl)-carbodiimide; ^(6)^ NHS, *N*-Hydroxysuccinimide.

**Table 2:** Summary of analytes, immobilized enzymes, immobilization methods, and LODs of impedimetric enzyme biosensors.

Analytes	Immobilized Enzymes	Immobilization Methods	LOD ^(8)^	Ref.
H_2_O_2_	CAT ^(1)^	Adsorption	0.025 nM	[73]
Glucose	GOx ^(2)^	Covalent bonding (EDC ^(4)^-NHS ^(5)^)	15.6 µM	[70]
	GOx	Adsorption	1 mM	[74]
Glutamate	GLOD ^(3)^	Cross-linking (GTA ^(6)^)	20 µM	[75]
Alcohol	Alcohol oxidase	Electrochemical polymerization (aniline)	–	[76]
Cyanide	Glycerol catalase	Photopolymerization (PVA-SbQ ^(7)^)	4 µM	[77]
Urea	Urease	Covalent bonding (Eudragit S-100, carbodiimide)	0.02 M	[78]
Diazinon	Lipase	Cross linking (GTA)	10 nM	[71]
Trichlorfon	Butyrylcholinesterase	Adsorption	0.1 ppm	[79]

^(1)^ CAT, Catalyst; ^(2)^ GOx, Glucose oxidase; ^(3)^ GLOD, Glutamate oxidase; ^(4)^ EDC, 1-Ethyl-3-(3-dimethylaminopropyl)-carbodiimide; ^(5)^ NHS, *N*-Hydroxysuccinimide; ^(6)^ GTA, Glutaraldehyde; ^(7)^ PVA-SbQ, Poly(vinyl alcohol) bearing photopolymerizable styrylpyridinium groups; ^(8)^ LOD, Limit of detection.

**Table 3:** The enthalpy changes for enzymatic catalysis.

Enzymes	Reactants	−ΔH (kJ·mol^−1^)
NADH dehydrogenase	NADH	225
β-Lactamase	Penicillin G	115
Catalase	Hydrogen peroxide	100
Glucose oxidase	Glucose	80–100
Hexokinase	Glucose	75
Lactate dehydrogenase	Sodium pyruvate	62
Urease	Urea	61
Cholesterol oxidase	Cholesterol	53
Uricase	Urate	49
Trypsin	Benzoyl-l-arginine amide	29

**Table 4:** Summary of analytes, immobilized enzymes, immobilization methods and detection range/LOD of QCM enzyme biosensors.

Analytes	Immobilized Enzymes	Immobilization Methods	Detection Range/LOD	Ref.
Acetylcholine	HRP ^(1)^, choline OD, AChE ^(2)^	Covalent bonding (DSP ^(7)^, GTA)	1 × 10^−5^ M	[128]
Carbaryl	AChE, ChE ^(3)^	Adsorption, Covalent bonding (Cystamine, GTA ^(8)^)	1.0 × 10^−7^ M	[127]
Dichlorvos		Adsorption	1 ppm	[129]
Paroxon	AChE	Adsorption, Covalent bonding (Cystamine, GTA)	5.0 × 10^−8^ M	[127]
Hydrogen peroxide	HRP or HRP/GOx ^(4)^	Covalent bonding (Cystamine, GTA)	0.13–80 µmol·L^−1^	[130]
Glucose			0.08–10 µmol·L^−1^	
Cholesterol	Cease ^(5)^, Cox ^(6)^, HRP	–	3 × 10^−4^ M	[131]
Urea	Urease	Adsorption, Cross linking (GTA)	0.2 mM	[125]
Gluconic acid	GOx	Adsorption	1.4–14.0 mM	[126]
Dimethyl methylphosphonate	AChE	Cross linking (GTA)	0–50 mg·m^−3^	[132]

^(1)^ HRP, Horseradish peroxidase; ^(2)^ AChE, Acetylcholinesterase; ^(3)^ ChE, cholinesterase; ^(4)^ GOx, Glucose oxidase; ^(5)^ CEase, Cholesterol esterase; ^(6)^ COx, Cholestrol oxidase; ^(7)^ DSP, Dithiobis(succimidylpropionate); ^(8)^ GTA, Glutaraldehyde.
